# Molecular epidemiological typing of *Neisseria gonorrhoeae* isolates identifies a novel association between genogroup G10557 (G7072) and decreased susceptibility to cefixime, Germany, 2014 to 2017

**DOI:** 10.2807/1560-7917.ES.2020.25.41.1900648

**Published:** 2020-10-15

**Authors:** Sebastian Banhart, Klaus Jansen, Susanne Buder, Thalea Tamminga, Sébastien Calvignac-Spencer, Tanja Pilz, Andrea Martini, Sandra Dudareva, Sergejs Nikisins, Kerstin Dehmel, Gabriele Zuelsdorf, Eva Guhl, Ingeborg Graeber, Peter K Kohl, Magnus Unemo, Viviane Bremer, Dagmar Heuer, on behalf of the GORENET study group

**Affiliations:** 1 Unit 'Sexually Transmitted Bacterial Infections', Department for Infectious Diseases, Robert Koch Institute, Berlin, Germany; 2 Unit 'HIV/AIDS, STI and Blood-borne Infections', Department for Infectious Disease Epidemiology, Robert Koch Institute, Berlin, Germany; 3 German Reference Laboratory for Gonococci, Department of Dermatology and Venerology, Vivantes Hospital Berlin, Berlin, Germany; 4 Project Group 'Epidemiology of Highly Pathogenic Microorganisms', Robert Koch Institute, Berlin, Germany; 5 Charité Universitätsmedizin Berlin, Berlin, Germany; 6 WHO Collaborating Centre for Gonorrhoea and Other STIs, Faculty of Medicine and Health, Örebro University, Örebro, Sweden; 7 The members of the GORENET study group are acknowledged at the end of the article

**Keywords:** *Neisseria gonorrhoeae*, antimicrobial resistance, resistance surveillance, molecular typing, NG-MAST, Cefixime, G1407, G10557 (G7072)

## Abstract

**Background:**

Emerging antimicrobial resistance (AMR) challenges gonorrhoea treatment and requires surveillance.

**Aim:**

This observational study describes the genetic diversity of *Neisseria gonorrhoeae* isolates in Germany from 2014 to 2017 and identifies *N. gonorrhoeae* multi-antigen sequence typing (NG-MAST) genogroups associated with AMR or some patient demographics.

**Methods:**

1,220 gonococcal isolates underwent AMR testing and NG-MAST. Associations between genogroups and AMR or sex/age of patients were statistically assessed.

**Results:**

Patients’ median age was 32 years (interquartile range: 25–44); 1,078 isolates (88.4%) originated from men. In total, 432 NG-MAST sequence types including 156 novel ones were identified, resulting in 17 major genogroups covering 59.1% (721/1,220) of all isolates. Genogroups G1407 and G10557 (G7072) were significantly associated with decreased susceptibility to cefixime (Kruskal–Wallis chi-squared: 549.3442, df: 16, p < 0.001). Their prevalences appeared to decline during the study period from 14.2% (15/106) to 6.2% (30/481) and from 6.6% (7/106) to 3.1% (15/481) respectively. Meanwhile, several cefixime susceptible genogroups’ prevalence seemed to increase. Proportions of isolates from men differed among genogroups (Fisher’s exact test, p < 0.001), being e.g. lower for G25 (G51) and G387, and higher for G5441 and G2992. Some genogroups differed relative to each other in affected patients’ median age (Kruskal–Wallis chi-squared:  47.5358, df:  16, p < 0.001), with e.g. G25 (G51) and G387 more frequent among ≤ 30 year olds and G359 and G17420 among ≥ 40 year olds.

**Conclusion:**

AMR monitoring with molecular typing is important. Dual therapy (ceftriaxone plus azithromycin) recommended in 2014 in Germany, or only the ceftriaxone dose of this therapy, might have contributed to cefixime-resistant genogroups decreasing.

## Introduction

Gonorrhoea is a sexually transmitted infection (STI) caused by the Gram-negative bacterium *Neisseria gonorrhoeae*. Estimations of numbers of persons with gonorrhoea worldwide by the World Health Organization (WHO) resulted in 86.9 million cases among adults aged 15 to 49 years in 2016 [1], making *N. gonorrhoeae* the third most common non-viral STI. In the European Union/European Economic Area (EU/EEA), the number of reported gonorrhoea cases has increased by > 200% since 2008. The highest incidence of cases is among young adults (15–24 years of age). During the 2013 to 2018 period, men who have sex with men (MSM) accounted for ca 25–30% of all cases in the EU/EEA; however, between 2009 and 2018, clear increases were also recorded among heterosexual men, men without sexual orientation reported, and women [2].

If left untreated, *N. gonorrhoeae* infections can cause severe reproductive tract complications and develop into disseminated infections [3-5]. Unfortunately, rapid development and spread of gonococcal antimicrobial resistance (AMR) limit options to control and treat the infection. Accordingly, the WHO lists *N. gonorrhoeae* as a high priority pathogen posing threat to human health due to development of AMR [6] and measures to strengthen AMR surveillance are recommended [7,8]. Particularly alarming is the emergence of strains clinically resistant to last-line antibiotics, such as extended-spectrum cephalosporins (ESCs; cefixime and ceftriaxone) and azithromycin, leading gonorrhoea to become a global public health concern [3,7,8].

In 2014, following the rise of cefixime resistance, the German STI Society (Deutsche STI-Gesellschaft, DSTIG) revised the national treatment guidelines from a first-line recommendation of cefixime monotherapy to a dual therapy including ceftriaxone and azithromycin. However, cefixime remains used, ideally after AMR testing, for example if parenteral administration of ceftriaxone is not available or refused [9]. In 2019, German treatment guidelines were adjusted towards a more individualised recommendation taking into account the patient’s compliance, the site of infection and/or the AMR profile of the isolated bacteria, so monotherapy with a high dose (1 g) of ceftriaxone is also recommended in specific circumstances [10]. 

Since 2017, cases of infections with multidrug- and extensively drug-resistant *N. gonorrhoeae* have been reported from several countries worldwide, including in the EU [11-14]. It is likely that those cases represent only a minor fraction of the real number of patients infected by these resistant gonococcal strains. In light of this AMR development, the WHO and the European Centre for Disease Prevention and Control (ECDC) emphasise the need to strengthen national surveillance systems for *N. gonorrhoeae* [7,8,15,16].

Currently, available data on AMR and epidemiology of *N. gonorrhoeae* in Germany are limited. Gonorrhoea has only been mandatorily reportable in the country since the beginning of 2020 and, so far, *N. gonorrhoeae* AMR data have been generated in cross-sectional studies performed in individual regions [17-20] and from the European Gonococcal Antimicrobial Surveillance Programme (Euro-GASP), where a total number of 1,193 isolates with information on AMR were submitted by the German Reference Laboratory for Gonococci between 2009 and 2018 [21]. Of these isolates, 11 (0.9%) showed resistance against ceftriaxone and 57 (4.8%) were resistant to cefixime. Resistance against azithromycin was detected in 58 isolates (4.9%).

To reinforce surveillance of AMR at a national level, the Gonococcal Resistance Network (GORENET), coordinated by the Robert Koch Institute (RKI) in cooperation with the German Reference Laboratory for Gonococci, was set up in 2013 (with data collection starting in 2014) in Germany [9,22]. Participation in the network was voluntary, with no financial compensation for involved laboratories. To obtain a better geographical coverage of the data, GORENET aimed to recruit laboratories from all regions of the country. To this effect, a mapping of all laboratories testing for *N. gonorrhoeae* in Germany was first performed by RKI. From laboratories in different regions, expressing interest in providing *N. gonorrhoeae* isolates with linked minimal patient data, private and hospital laboratories with a wider catchment area and a higher number of *N. gonorrhoeae* tests were prioritised for inclusion in GORENET. Within GORENET, molecular typing of gonococcal isolates using *N. gonorrhoeae* multi-antigen sequence typing (NG-MAST) is performed [23]. In conjunction with data from AMR testing, this allows to characterise the gonococcal population in Germany, identify strains linked to AMR and describe associations with minimal epidemiological characteristics (sex and age) of patients.

In 2018, a report on AMR of *N. gonorrhoeae* in Germany was published on the basis of isolates collected within GORENET between 2014 and 2015 [9]. The report also described that GORENET had reached a relatively even geographical representation of all regions with a slightly reduced coverage of central and southern Germany. In this study involving a total of 537 isolates, nine (1.7%) isolates were resistant to cefixime and 58 (10.8%) showed resistance against azithromycin [9]. Among all isolates collected, none with resistance against ceftriaxone were identified [9]. 

In the current study, we describe gonococcal isolates (n = 1,220) collected from 2014 to 2017 in the frame of GORENET. Results from molecular typing (NG-MAST) are analysed in combination with phenotypic and minimal epidemiological data. This allows us to further describe the diversity of the gonococcal population in Germany and to identify *N. gonorrhoeae* strains and NG-MAST genogroups associated with AMR or sex or age of patients.

## Methods

### Isolate and data collection

Between January 2014 and December 2017, *N. gonorrhoeae* isolates from patient samples and patient-related data were collected by GORENET laboratories and submitted to the German Reference Laboratory for Gonococci. Laboratories voluntarily participating in the GORENET were selected as previously described [9]. Submitted data included sample identification number, date of sampling, as well as sex and year of birth of the patient. In case of an unknown date of sampling, the date of sample receipt at the reference laboratory was used for further analyses. Finally, plausibility checks were performed on all reported data. These included checking for duplicates and data discrepancies by cross-tabulating variables with the same information from all different data sources (data directly reported from GORENET laboratories, data from central retesting at the German Reference Laboratory for Gonococci and data from paper laboratory sheets accompanying submitted isolates). In cases in which data were inconclusive (e.g. incorrect year of birth) data were cross-checked with the paper laboratory sheets and corrected on basis of these sheets.

### Antimicrobial susceptibility testing

All isolates submitted to the reference laboratory for AMR testing were initially cultured by the participating GORENET cooperation laboratories. At the reference laboratory, all isolates were cultured on non-selective agar medium and identification of *N. gonorrhoeae* was performed by detection of oxidase-positive, Gram-negative diplococci with typical colony morphology and using Phadebact Monoclonal GC test (MKL Diagnostics AB, Sollentuna, Sweden). To verify inconclusive results, API-NH (bioMérieux SA, Marcy l'Etoile, France) was performed. After species identification, minimum inhibitory concentrations (MICs) of azithromycin, benzylpenicillin, cefixime, ceftriaxone and ciprofloxacin were determined by Etest (bioMérieux SA, Marcy l'Etoile, France) according to the manufacturer’s instructions [9]. To be consistent with AMR data published for other countries in a similar sampling period, results were interpreted according to the clinical breakpoints valid at the end of the sampling period stated by the European Committee on Antimicrobial Susceptibility Testing (EUCAST) [24].

### Molecular epidemiological typing

For molecular epidemiological typing, NG-MAST was performed [23]. In the start-up years 2014 and 2015, a subset of isolates submitted to the German Reference Laboratory for Gonococci was chosen for NG-MAST at the RKI on the basis of regional information of submitting physicians or laboratories. By this, we aimed to reach wide geographical representation of isolates in Germany, however, without being proportional to the number of isolates submitted from each region. In 2016 and 2017, extended genotyping capacities allowed us to subject all submitted isolates to NG-MAST. DNA extractions of *N. gonorrhoeae* isolates were performed as previously described [23]. Internal fragments of *porB* and *tbpB* were PCR amplified and sequenced using the previously published primers *por* forward, *por* reverse, *tbpB* forward, and *tbpB* reverse and protocol, with minor modifications (http://www.ng-mast.net/misc/info2.asp). Sequencing of both DNA strands was performed to improve accuracy.

### Assignment of NG-MAST sequence types and genogroups

NG‐MAST sequence types (STs) were assigned using the global NG‐MAST database (http://www.ng-mast.net). For the most frequently observed STs (represented by 10 isolates or more), NG-MAST genogroups were assigned as previously reported [15,16]. Genogroups were named using the letter G followed by the number of the predominant ST within each group. As the predominant ST in a given genogroup can vary based on the analysed sample set, genogroup names might be different in comparable studies. To account for this, we added previously published genogroup names in brackets, e.g. G10557 (G7072).

### Multiple sequence alignment and phylogenetic analysis

To allow for the visualisation of *N. gonorrhoeae* sequence diversity, we first analysed concatenated sequences of NG-MAST trimmed *porB* and *tbpB* sequences. The concatenated *porB* and *tbpB* sequence from *N. meningitidis* strain MC58 (GenBank accession number: NC_003112) was included as outgroup. Sequences were aligned with multiple sequence comparison by log-expectation (MUSCLE) [25] and the resulting alignment (1,221 sequences and 1,091 positions) was used to build a phylogenetic tree based on Tamura–Nei distances using the neighbour-joining (NJ) method [26] as implemented in Geneious v11.1.5 [27]. The resulting dendrogram was annotated using the online platform iTOL [28]. Colour gradients for the visualisation of MICs were set to white for 0 mg/L and to blue for the EUCAST clinical breakpoint for resistance [24].

While this first tree allowed for an immediate and natural display of the assigned STs and genogroups, it was not amenable to rigorous evolutionary investigations of AMR emergence in *N. gonorrhoeae*. Using the same alignment of concatenated sequences, we therefore ran complementary analyses using a combination of explicit modelling of phenotype distribution change across maximum likelihood (ML) phylogenetic trees. For a detailed description of these analyses see Supplementary Methods S1.

### Statistical analyses

For categorical variables, absolute and relative frequencies were determined. For continuous variables (age), median and interquartile range (IQR) were calculated. For graphical representation of MICs, geometric means and 95% confidence intervals were determined.

For continuous or ordered variables (age and MICs for cefixime, ceftriaxone and azithromycin), we ran a Kruskal–Wallis rank sum test to compare genogroup mean ranks and a post-hoc Dunn test with Benjamini–Hochberg correction for multiple testing to assess individual genogroup pairwise comparisons with the R packages *dunn.test*. To improve the readability of the results of these multiple comparisons, the p value table was converted to compact letter display using the R package *rcompanion*. As an output, genogroups are separated by letters. Genogroups sharing a letter are not significantly different.

To investigate whether sex and genogroup were independent, we first tabulated a contingency table using the R package *MASS*. Since the contingency table comprised cells with small or zero values, we then applied a Fisher’s exact test, computing p values by Monte Carlo simulations (using 10,000 replicates) with the R package *stats*.

For all tests, the statistical significance threshold was set a priori to 0.05. We ran all analyses using R version 3.6.0 (26 April 2019) [29].

### Ethical statement

The protocol of data collection was verified by the data protection officer at the RKI. No additional approval from an ethics committee was considered to be necessary as the study complies with the national guidelines according to the German Data Protection Act and no patient-identifying data were collected.

## Results

### *Neisseria gonorrhoeae* isolates and gonorrhoea patients

In total, 1,220 isolates underwent NG-MAST (106 from 2014, 122 from 2015, 511 from 2016, and 481 from 2017). Of these 1,220 isolates, 88.4% (n = 1,078) were obtained from men, 11.2% (n = 137) from women, and 0.4% (n = 5) from patients not reporting sex. The median age was 32 years (IQR: 25–44) overall, 33 years (IQR: 26–44) for men and 28 years (IQR: 23–41) for women.

### Frequency of NG-MAST sequence types

A total of 432 different STs including 156 novel STs were identified, comprising 342 different *porB* alleles and 99 different *tbpB* alleles. Of all detected unique STs, 148 were shared by ≥ 2 isolates, covering 76.6% (n = 935) of all 1,220 typed isolates. The STs with a prevalence of ≥ 1% were ST2992 (4.5%; n = 55), ST5441 (3.9%; n = 47), ST5624 (3.2%; n = 39), ST25 (3.0%; n = 37), ST387 (2.5%; n = 30), ST1407 (2.4%; n = 29), ST2400 (2.4%; n = 29), ST359 (2.3%; n = 28), ST9184 (2.3%; n = 28), ST10557 (2.3%; n = 28), ST11461 (2.3%; n = 28), ST13489 (1.4%; n = 17), ST9208 (1.3%; n = 16), ST13878 (1.2%; n = 15), ST5793 (1.1%; n = 14), ST2318 (1.1%; n = 13), ST17420 (1.1%; n = 13), and ST225 (1.1%; n = 13) (Figure S1A, Table S1).

### Frequency of NG-MAST genogroups

Overall, 722 isolates were grouped into 17 genogroups, covering 59.2% of all 1,220 typed isolates. Of these, genogroup G1405 was the only genogroup consisting of only one ST (ST1405). The prevalence of determined genogroups was as follows: G2400 (6.8%; n = 83), G1407 (6.7%; n = 82), G5441 (6.2%; n = 76), G25 (G51) (5.6%; n = 68), G2992 (5.5%; n = 67), G10557 (G7072) (5.3%; n = 65), G11461 (3.6%; n = 44), G5624 (3.4%; n = 41), G387 (3.3%; n = 40), G359 (2.5%; n = 31), G17420 (2.1%; n = 26), G2318 (1.7%; n = 21), G4186 (G9909) (1.6%; n = 19), G9208 (1.4%; n = 17), G5793 (1.2%; n = 15), G225 (1.1%; n = 14), and G1405 (1.0%; n = 12) (Figure S1B, Table S2).

### NG-MAST genogroups and sex and age of corresponding gonorrhoea patients

Age and sex were reported for 99.8% (1,217/1,220) and 99.6% (1,215/1,220) of all isolates, respectively. First, we compared the overall proportion of males (88.4%; n = 1,078) in the years observed with the proportion of males infected with *N. gonorrhoeae* of a given genogroup (Figure S2A). Here, we detected clear differences between groups of patients infected with *N. gonorrhoeae* of different genogroups (Fisher’s exact test, p value < 0.001). For example, genogroups G25 (G51), G387, G17420, G225, and G1405 showed lower proportion of males with 48 men (total n = 68), 31 men (total n = 40), 20 men (total n = 26), 11 men (total n = 14), and nine men (total n = 12), respectively (Figure S2A). In contrast, other genogroups had higher proportion of males, such as G5441 (75/76), G2992 (64/67), G11461 (43/44), G5624 (38/41), G9208 (17/17), and G5793 (15/15) (Figure S2A).

Next, we compared the overall median age (32 years; IQR: 25–44) with the median age of patients infected with *N. gonorrhoeae* of different genogroups (Figure S2B). Here, we also detected clear differences between genogroups (Kruskal–Wallis chi-squared:  47.5358, df:  16, p value < 0.001), with some preferentially circulating among people aged 30 years or younger, for example G25 (G51) (28 years, IQR: 23–36, n = 68) and G387 (27 years, IQR: 23–43, n = 40) (Figure S2B). Other genogroups were predominantly found in people aged 40 years or older, for example G359 (45 years, IQR: 31–54, n = 31) and G17420 (50 years, IQR: 31–57, n = 26) (Figure S2B).

### NG-MAST genogroups and antimicrobial resistance associations

**Figure 1:**
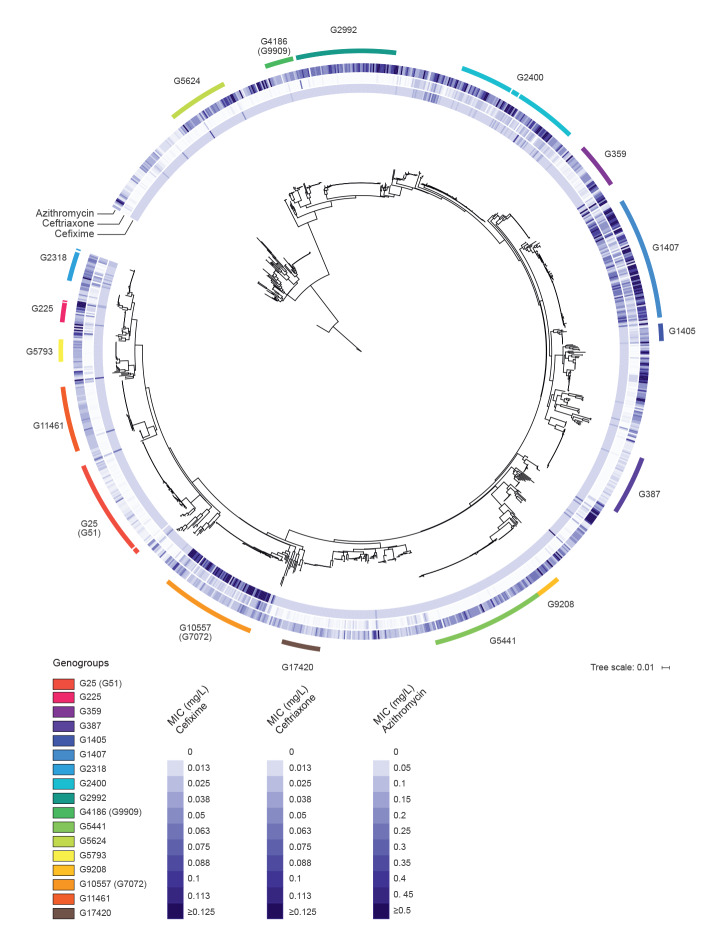
Neighbour-joining tree based on concatenated *Neisseria gonorrhoeae* *porB* and *tbpB* sequences^a^ derived from patients’ isolates, Germany, 2014–2017 (n = 1,220 isolates)

MIC: minimum inhibitory concentration; NG-MAST: *Neisseria gonorrhoeae* multi-antigen sequence typing.

^a^ The neighbour-joining tree is based on Tamura–Nei distances in a multiple sequence alignment of concatenated *porB* and *tbpB* sequences.

**Figure 2:**
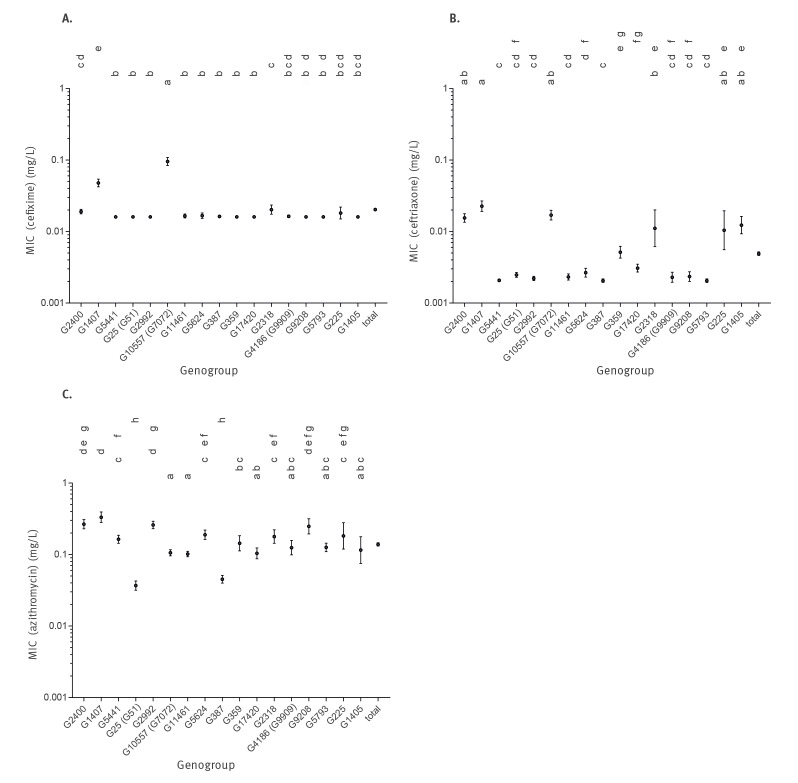
Minimum inhibitory concentrations of (A) cefixime, (B) ceftriaxone and (C) azithromycin, by assigned *Neisseria gonorrhoeae* multi-antigen sequence typing genogroups

EUCAST: European Committee on Antimicrobial Susceptibility Testing; MIC: minimum inhibitory concentration.

To visualise associations of AMR with certain genogroups, an NJ phylogenetic tree derived from corrected distances in an alignment of concatenated *porB* and *tbpB* sequences was first overlaid with colour-coded MIC values obtained from AMR testing for the therapeutically relevant antibiotics cefixime, ceftriaxone and azithromycin (Figure 1). This identified genogroups G10557 (G7072) and G1407 as significantly associated with a decreased susceptibility to cefixime (Kruskal–Wallis chi-squared:  549.3442, df:  16, p value < 0.001) (Figure 1, Figure 2A). Genogroup G10557 (G7072) accounted for nine of all 13 cefixime-resistant isolates collected (Figure S3, Table S3). Geometric means of MIC values for G10557 (G7072) and G1407 were 0.096 mg/L and 0.048 mg/L, respectively (Figure 2A). For G10557 (G7072), this is close to the EUCAST clinical breakpoint for cefixime resistance (MIC > 0.125 mg/L) [24]. For ceftriaxone, no significant associations could be detected; however, moderately elevated mean MIC values were seen for G2400, G1407 and G10557 (G7072) (Figure 1) ranging from 0.015 to 0.022 mg/L (Figure 2B). For azithromycin, all genogroups except for G25 (G51) and G387 showed only slightly elevated mean MIC values from 0.102 to 0.333 mg/L (Figure 2C).

The phylogenetic tree is overlaid with the assigned NG-MAST genogroups and the MICs of cefixime, ceftriaxone and azithromycin (all colour-coded). Tree scale shows substitutions per site.

MICs are shown as geometric means with 95% confidence intervals plotted against genogroups. Letters indicate compact lettering translation of the Dunn test. Genogroups sharing a letter are not significantly different. EUCAST clinical resistance breakpoints are MIC > 0.125 mg/L (cefixime, ceftriaxone) and MIC > 0.5 mg/L (azithromycin).

We assessed the statistical robustness of these observations using a combination of explicit modelling of phenotype distribution change across ML phylogenetic trees reconstructed independently for *porB* and *tbpB* (only for azithromycin and cefixime) and univariate statistical tests (for azithromycin, cefixime and ceftriaxone). Bayes factor comparison of models involving changes and null models assuming homogenous phenotype distribution across *N. gonorrhoeae* phylogenetic trees clearly favoured a heterogeneous phenotype distribution, with ca one change inferred for cefixime and five for azithromycin (Table S4).

In line with this, univariate analyses supported significantly different mean MIC ranks across genogroups for cefixime, ceftriaxone and azithromycin (cefixime: Kruskal–Wallis chi-squared:  549.3442, df:  16, p value < 0.001; ceftriaxone: Kruskal–Wallis chi-squared:  527.0183, df:  16, p value < 0.001; azithromycin: Kruskal–Wallis chi-squared:  428.5228, df:  16, p value < 0.001). Dunn tests confirmed the statistical significance of all genogroup-specific phenotype associations discussed above (Figure 2).

Genogroups associated with decreased susceptibility to benzylpenicillin and ciprofloxacin are shown in Figure S4A and B, respectively.

Taken together, these observations demonstrate that specific genogroups and evolutionary lineages are associated with elevated MIC values and highlight the potential of molecular epidemiological typing to identify these genogroups from the background of isolates generally tested for AMR. Moreover, our analysis revealed G10557 (G7072) as a genogroup significantly associated with decreased susceptibility to cefixime.

### NG-MAST genogroups and changes in prevalence

**Figure 3:**
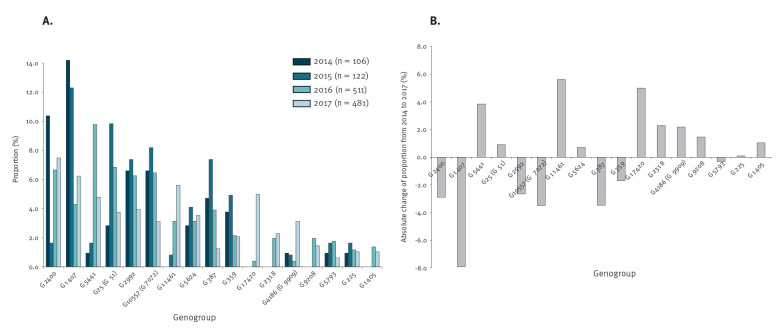
(A) Annual proportion of isolates within assigned NG-MAST genogroups and (B) the absolute change of proportions in the study period, Germany, 2014–2017

To describe the relative stability of the gonococcal population in Germany, we calculated the prevalence of individual genogroups circulating each year to check for any apparent changes over the 2014 to 2017 period. The three genogroups which seemed to most prominently decrease in prevalence from 2014 to 2017 were G1407 (from 14.2% (15/106) to 6.2% (30/481)), G10557 (G7072) (from 6.6% (7/106) to 3.1% (15/481)) and G387 (from 4.7% (5/106) to 1.2% (6/481)) (Figure 3A, B). Interestingly, two of these genogroups with decreasing prevalence were associated with elevated MIC values for cefixime and ceftriaxone (G1407, G10557 (G7072); Figure 1). Genogroups with an apparent increase in prevalence from 2014 to 2017 included G11461 (from 0.0% (0/106) to 5.6% (27/481)), G17420 (from 0.0% (0/106) to 5.0% (24/481)) and G5441 (from 0.9% (1/106) to 4.8% (23/481)), all of which had low MIC values for cefixime, ceftriaxone and azithromycin (Figure 2, 3).

NG-MAST: *Neisseria gonorrhoeae* multi-antigen sequence typing.

## Discussion

This is the first study to genetically describe the population of *N. gonorrhoeae* strains detected in Germany over a 4-year period of time, linking STs with AMR profiles and epidemiological data on age and sex. It provides evidence of a genetically diverse and dynamic gonococcal population in the country and shows an association between genogroup G10557 (G7072) and decreased susceptibility to cefixime, which had not been prior reported.

GORENET monitors antimicrobial susceptibility of *N. gonorrhoeae* in Germany and reported high rates of resistance against azithromycin, benzylpenicillin and ciprofloxacin in vitro between 2014 and 2015, while more than 98% of all strains remained susceptible to ceftriaxone and cefixime [9]. GORENET has reached a relatively even geographical representation for Germany; however, due to the lack of data on total numbers of *N. gonorrhoeae* AMR tests being performed in different regions of Germany, we were unable to adjust this representation proportional to the number of isolates submitted from each region. To identify subpopulations linked to AMR, isolates collected in the framework of GORENET additionally underwent NG-MAST and were assigned to genogroups consisting of genetically related STs. This analysis revealed that both G2400 and the multidrug resistance associated G1407 [15,16], were the most prevalent genogroups in Germany between 2014 and 2017. High proportions of these genogroups have also been detected in many other EU/EEA countries including Belgium, Hungary, Norway, Portugal, Slovenia, Spain and Switzerland, which might indicate putative transmissions [15,16,30]. Moreover, our study describes the gonococcal population in Germany as genetically diverse, which is in line with observations made worldwide [15,16,31,32].

Some genogroups have been associated with sex and distinct sexual networks. As we have considerably less data on women, and we do not have data on sexual orientation available, we cannot conclude about potential important ways of transmission on national and international level. Nevertheless, we observed lower proportions of males for G25 (G51) and G387 indicating that these strains might be more strongly associated with heterosexual contacts, whereas G5441 and G2992 were primarily isolated from men suggesting that these genogroups might predominantly circulate in networks of MSM. Previous European surveys linking genogroups with data on sexual orientation support the possible association of G25 (G51) and G387 with heterosexual individuals, while G2992 is described to be mainly present among MSM [15,16,33]. For G1407, an association with MSM in Europe was observed in 2009 and 2010, which then shifted to a stronger association with heterosexuals in 2013 [15,16,34]. Our data from 2014 to 2017 in Germany did not show a very high proportion of males carrying G1407 isolates, which potentially further supports the change in the epidemiological properties of this genogroup towards a more pronounced distribution via heterosexual contacts [15].

Another epidemiological feature associated with genogroups is the patient’s age. For G25 (G51) and G387, we observed median ages below 30 years, whereas G359 and G17420 were mainly detected in people aged 40 years or older. Again, this observation is supported by previously published data [16].

In general, the overall median age of 32 years in our study is comparable to other surveys [15,16,31]. The proportion of gonococcal isolates from female patients in our study was approximately 11% which is similar to other reports [15,16,31] and likely indicates a sampling bias towards isolates from men and a larger spread among MSM. This bias and the small sample size for some genogroups affect the interpretation and thus, all described associations should be interpreted with caution.

Until 2019, national guidelines in Germany recommended treatment of *N. gonorrhoeae* infections by dual therapy combining an ESC (ceftriaxone or cefixime) with azithromycin [9]. Here, we detected associations of G1407 and G10557 (G7072) with elevated MICs for cefixime, threatening therapy options with cefixime and other oral ESCs. Occasionally, occurrence of STs contributing to this genogroup have been reported, such as ST7072 in Spain, Norway and Germany [35-37], ST10557 in Slovakia [37], and ST13876 in Canada [38], with in some cases, detection of reduced susceptibility to cefixime in ST7072 [35,36]. To our knowledge, this is the first study to link genogroup G10557 (G7072) with decreased susceptibility to cefixime. Interestingly, G10557 (G7072) accounted for nine of all 13 reported cefixime resistant isolates in the present study, suggesting that G10557 (G7072) is the predominant genogroup causing infections with cefixime-resistant isolates in Germany.

The prevalence of the multidrug-resistant genogroup G1407 and cefixime-resistant genogroup G10557 (G7072) declined during the observation period and seemed to be replaced by new genogroups that showed a higher susceptibility to ESCs. Yet, these conclusions might be influenced by different sample sizes in 2014–2015 compared with 2016–2017. A decline in G1407 prevalence between 2009 and 2013 has also been reported in Europe [15]. Reasons for this reduction might include the introduction of a dual therapy combining ceftriaxone and azithromycin, increased testing for *N. gonorrhoeae* especially in extra-urogenital sites and possibly a reduced fitness of these resistant strains compared with other gonococcal strains [39-41]. In contrast, genogroups showing the most prominent increase from 2014 to 2017 (G5441, G11461 and G17420) seem to be fully susceptible to both cefixime and ceftriaxone, emphasising the complex population dynamics of *N. gonorrhoeae* in Germany. This underlines the importance of monitoring AMR in combination with molecular epidemiological typing, nationally and internationally.

## The GORENET study group

Thomas Back (Labour Schottdorf MVZ GmbH, Augsburg), Anja Berger (Bayerisches Landesamt für Gesundheit und Lebensmittelsicherheit, Oberschleißheim, Sachgebiet GE 2.2 Infektiologie, Humanbakteriologie, Mykologie und Konsiliarlabor Diphtherie), Valerie Chapot, Jörg Steinmann (Institut für Medizinische Mikrobiologie, Universitätsklinikum Essen), Stephan Eicke (Labour Dr Eicke, Berlin), Claudia Friedrichs (Medizinisches Labour Ostsachsen MVZ GbR, Görlitz), Andreas Groß, Hans-Jochen Hagedorn (Labour Krone, Bad Salzuflen), Alexander Halfmann (Institut für Medizinische Mikrobiologie und Hygiene, Universitätsklinikum des Saarlandes, Homburg), Britt Hornei (MVZ Synlab Leverkusen GmbH, Leverkusen), Ralf Ignatius (Labour Enders und Kollegen MVZ, Stuttgart), Simone Korten, Hany Sahly (Labour Lademannbogen MVZ GmbH, Hamburg), Elzbieta Kozub-Witkowski (LADR GmbH Medizinisches Versorgungszentrum Dr Kramer and Kollegen, Geesthacht), Sabine Krämer (MVZ Labour Eveld and Kollegen, Essen), Margit Kühn (Labour Becker, Olgemöller und Kollegen, München), Anke Liebetrau (MVZ Labour Dr Reising-Ackermann und Kollegen, Leipzig), Thomas Meyer (Institut für Medizinische Mikrobiologie, Virologie und Hygiene, Universitätsklinikum Hamburg-Eppendorf), Klaus Oberdorfer (Labour Dr Limbach und Kollegen, Heidelberg), Roland Pfüller (MDI Laboratorien GmbH, Berlin), Caroline Ruckert (MVZ für Laboratoriumsmedizin und Mikrobiologie Koblenz-Mittelrhein, Koblenz), Roman Schwarz (Labour Mönchengladbach, Medizinisches Versorgungszentrum Dr Stein + Kollegen, Limbach Gruppe), Daniela Walch, Madeleine Mai (Institut für Labormedizin, Mikrobiologie und Krankenhaushygiene, Krankenhaus Nordwest, Frankfurt am Main), Thomas A. Wichelhaus (Institut für Medizinische Mikrobiologie und Krankenhaushygiene, Universitätsklinikum Frankfurt, Goethe-Universität), Hilmar Wisplinghoff (Labour Dr Wisplinghoff, Köln), Nicole Wüppenhorst (Institut für Hygiene und Umwelt, Abteilung Medizinische Mikrobiologie, Hamburg).

## Supplementary Data

2884000 Supplementary Material
