# Supplementary material for: Molecular epidemiological typing of Neisseria gonorrhoeae isolates identifies a novel association between genogroup G10557 (G7072) and decreased susceptibility to cefixime, Germany, 2014 to 2017
Source: Euro Surveill. 2020 Oct 15;25(41):1900648. doi: 10.2807/1560-7917.ES.2020.25.41.1900648 (PMC7565851; doi:10.2807/1560-7917.ES.2020.25.41.1900648)
Supplement: Supplementary Material [file 1900648_Supplement.pdf]

Supplementary Material

This supplementary material is hosted by *Eurosurveillance* as supporting information alongside the article "Molecular epidemiological typing of Neisseria gonorrhoeae isolates identifies a novel association between genogroup G10557 (G7072) and decreased susceptibility to cefixime, Germany, 2014 to 2017", on behalf of the authors, who remain responsible for the accuracy and appropriateness of the content. The same standards for ethics, copyright, attributions and permissions as for the article apply. Supplements are not edited by *Eurosurveillance* and the journal is not responsible for the maintenance of any links or email addresses provided therein.

Supplementary Methods S1

Multiple sequence alignment and phylogenetic analysis

Using the same alignment of concatenated sequences, we ran complementary analyses. After reduction of the sequence set to unique sequences using Fabox v1.5 [1], we selected conserved blocks of the alignment using gblocks [2] as implemented in Seaview v4 [3]. The alignment was further reduced to sequences exhibiting a maximum 97% identity using T-Coffee [4] which we examined for evidence of recombination using the implementation of GARD [5] on the Datamonkey Adaptive Evolution Server [6]. This analysis identified a single recombination breakpoint at the junction of *porB* and *tbpB* conserved blocks. We therefore conducted all following analyses on the two separate alignments. For these phylogenetic reconstructions we used the complete set of sequences (as opposed to unique sequences) from which we removed 6 sequences which presented *N. meningitidis*-like *porB* and/or *tbpB* sequences; the final alignments comprised 1215 sequences and 372 (*porB*) or 337 positions (*tbpB*). We ran maximum likelihood (ML) analyses using PhyML v3 [7] and the smart model selection procedure [8]. The Bayesian information criterion was used to select models of nucleotide evolution, tree search was performed using subtree pruning and regrafting and branch support was assessed using Shimodaira-Hasegawa-like approximate likelihood ratio tests (SH-like aLRT). We removed tips for which phenotype info was missing using the R package *ape* and then investigated whether AMR phenotype distribution changed throughout the tree using TreeBreaker [9]. TreeBreaker can currently only handle discrete characters. Accordingly we were unable to run this analysis on ceftriaxone resistance since only a single ceftriaxone resistant isolate was detected in the study. We determined Bayes factors comparing models with phenotype changes to a null model assuming no change and we extracted statistics on the number of changes from the TreeBreaker outfile using the R script provided by the authors on their github (https://github.com/ansariazim/treeBreaker). Following Kass and Raftery (1995), we considered 2 ln $BF_{12} > 10$ as showing decisive support for model 1 (in our case models involving phenotype distribution change) [10].

References

1. Villesen P. FaBox: an online toolbox for FASTA sequences. Mol Ecol Notes. 2007;7(6):965-8.
2. Talavera G, Castresana J. Improvement of phylogenies after removing divergent and ambiguously aligned blocks from protein sequence alignments. Syst Biol. 2007;56(4):564-77.
3. Gouy M, Guindon S, Gascuel O. SeaView Version 4: A Multiplatform Graphical User Interface for Sequence Alignment and Phylogenetic Tree Building. Molecular Biology and Evolution. 2010;27(2):221-4.
4. Notredame C, Higgins DG, Heringa J. T-Coffee: A novel method for fast and accurate multiple sequence alignment. J Mol Biol. 2000;302(1):205-17.
5. Pond SLK, Posada D, Gravenor MB, Woelk CH, Frost SDW. Automated phylogenetic detection of recombination using a genetic algorithm. Molecular Biology and Evolution. 2006;23(10):1891-901.
6. Weaver S, Shank SD, Spielman SJ, Li M, Muse SV, Pond SLK. Datamonkey 2.0: A Modern Web Application for Characterizing Selective and Other Evolutionary Processes. Molecular Biology and Evolution. 2018;35(3):773-7.
7. Guindon S, Dufayard JF, Lefort V, Anisimova M, Hordijk W, Gascuel O. New Algorithms and Methods to Estimate Maximum-Likelihood Phylogenies: Assessing the Performance of PhyML 3.0. Syst Biol. 2010;59(3):307-21.
8. Lefort V, Longueville JE, Gascuel O. SMS: Smart Model Selection in PhyML. Molecular Biology and Evolution. 2017;34(9):2422-4.
9. Ansari MA, Didelot X. Bayesian Inference of the Evolution of a Phenotype Distribution on a Phylogenetic Tree. Genetics. 2016;204(1):89-98.
10. Kass RE, Raftery AE. Bayes Factors. J Am Stat Assoc. 1995;90(430):773-95.

A

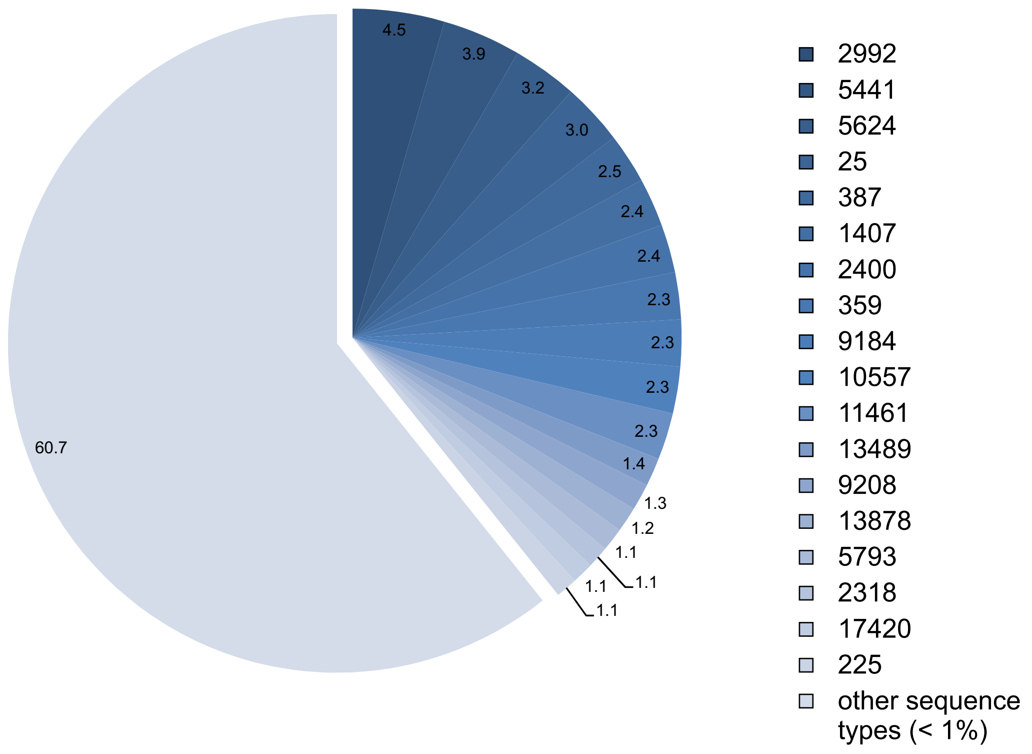

B

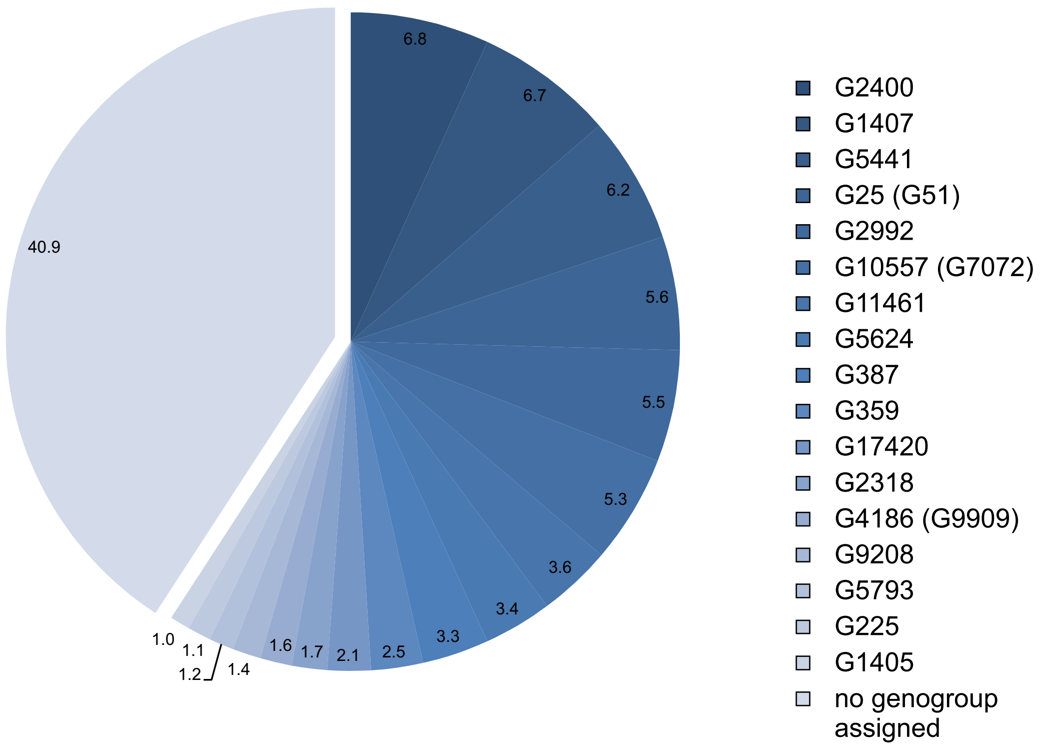

Supplementary Figure S1. Distribution of assigned NG-MAST sequence types **(A)** and assigned NG-MAST genogroups **(B)**, 2014 to 2017 in Germany.

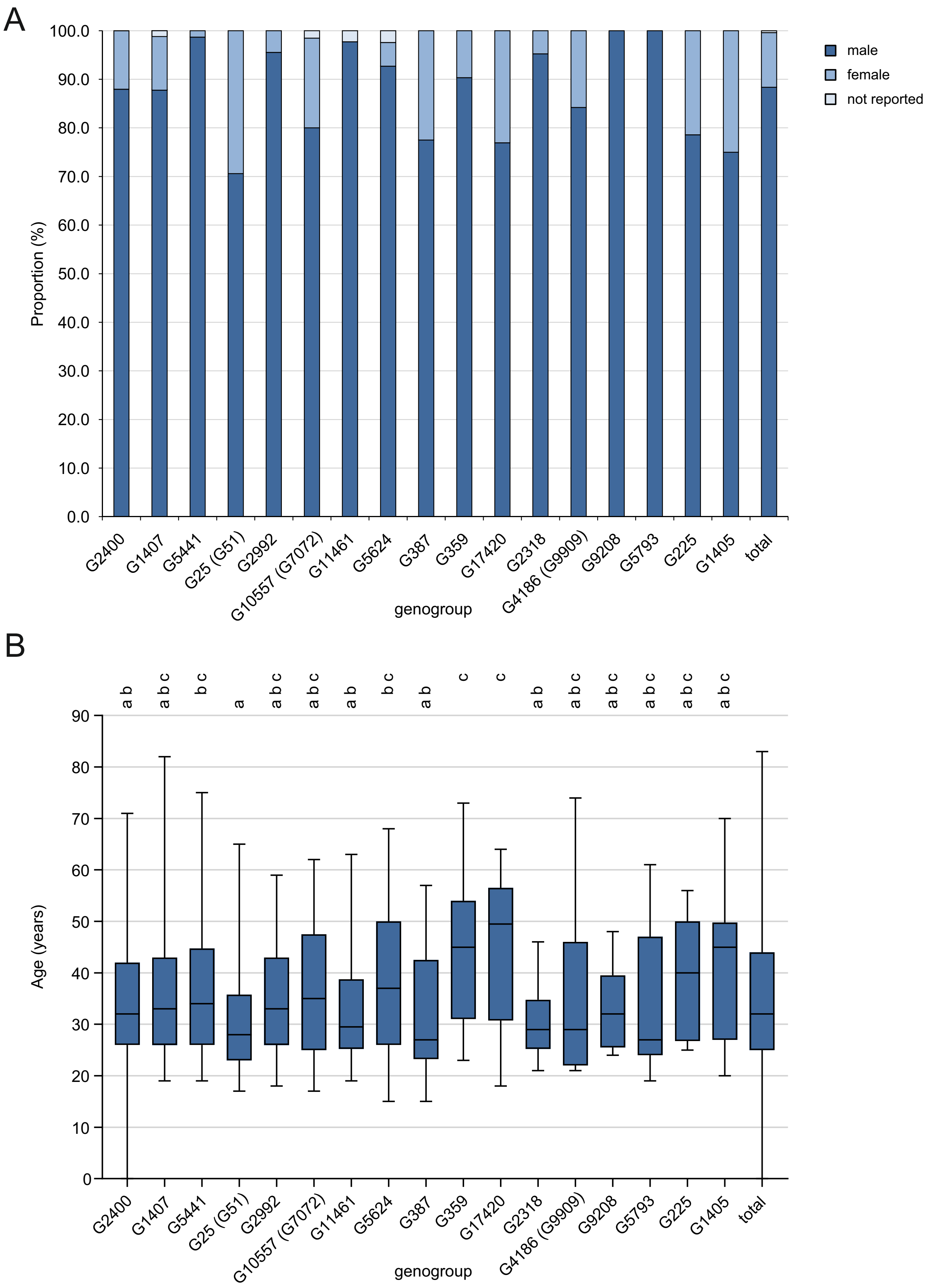

Supplementary Figure S2. Distribution of sex **(A)** and age **(B)**, by assigned NG-MAST genogroups. Median age, IQR, maximum and minimum is shown. Letters indicate compact lettering translation of the Dunn test. Genogroups sharing a letter are not significantly different.

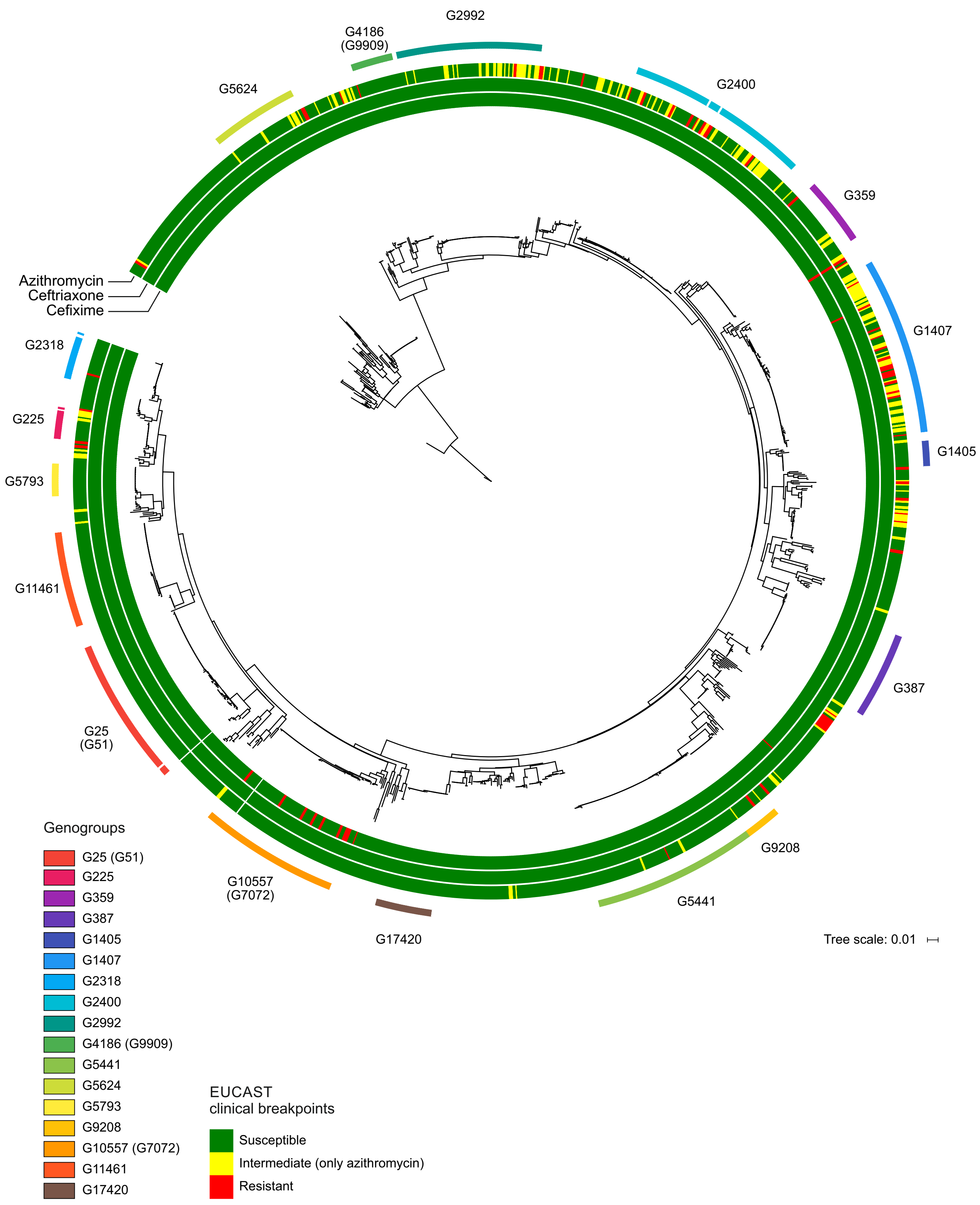

Supplementary Figure S3. Neighbor-Joining tree based on corrected distances in a multiple sequence alignment of concatenated porB and tbpB sequences representing 1220 isolates from patient samples collected between 2014 and 2017 in Germany. The phylogenetic tree is overlaid with the assigned NG-MAST genogroups and the EUCAST clinical breakpoints for cefixime, ceftriaxone and azithromycin (all colour-coded). Tree scale shows substitutions per site.

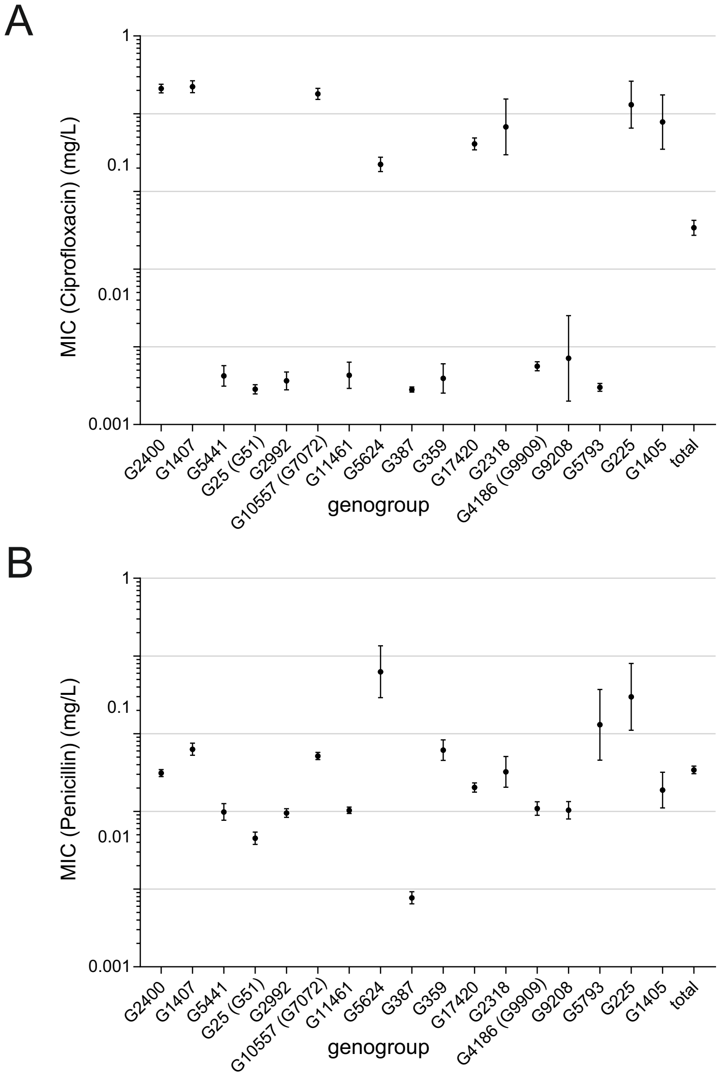

Supplementary Figure S4: MICs of ciprofloxacin **(A)** and penicillin **(B)**, by assigned NG-MAST genogroups. MICs are shown as geometric means with 95% confidence intervals plotted against genogroups. EUCAST clinical resistance breakpoints are 0.06 mg/L (ciprofloxacin) and 1 mg/L (penicillin).

Supplementary Table S1. Number of isolates per NG-MAST sequence type, Germany, 2014-2017 (n = 1220).

No. of isolates	Sequence types								
55	2992								
47	5441								
39	5624								
37	25								
30	387								
29	1407	2400							
28	359	9184	10557	11461					
17	13489								
16	9208								
15	13878								
14	5793								
13	225	2318	17420						
12	1405								
11	3779	14764	16076						
10	4186								
9	995	11581							
8	645	4995	11099						
7	292	9918	11755	17412	17615				
6	5785	17405							
5	1582	3346	3785	7072	7445	10789	12302	13873	14994
4	51	2212	3378	4244	4943	5533	6974	7272	10121
	11101	11421	12722	13876	17388	17497			
3	298	1612	1937	2997	3287	3935	4405	4706	5743
	6701	10082	10386	10421	10806	11746	12280	12547	12728
	13231	15199	17345	17413	17440	17450	17477	17480	17485
	17515	17612	17616						
2	5	26	273	865	1100	1993	3558	3588	4269
	5049	5341	5384	5985	6961	7381	7574	8016	8503
	9873	9909	9936	9972	10191	10193	10784	11432	12724
	12727	12737	13299	13862	13868	14051	15289	15726	16819
	17370	17390	17400	17403	17409	17410	17425	17443	17447
	17457	17458	17462	17478	17487	17491	17493	17495	17496
	17499	17505	17511	17621	18193				
1	21	28	210	217	355	356	433	508	615
	758	963	972	1128	1152	1288	1464	1503	1766
	1833	1861	1929	2051	3274	3519	3997	4189	4248
	4321	4654	4730	4734	4822	4925	4954	4977	5016
	5060	5119	5219	5223	5435	5445	5544	5570	5592
	5622	5656	6327	6339	6360	6955	6965	7221	7268
	7437	7638	7741	8009	8032	8058	8115	8241	8271
	8379	8380	8631	9153	9249	9382	9713	9869	9925
	10083	10107	10159	10215	10251	10419	10521	10800	11072
	11078	11083	11522	11525	11576	11602	11603	11661	11961
	12001	12331	12631	12721	12723	12725	12726	12729	12730
	12814	12821	13198	13470	13487	13491	13599	13732	13787
	13849	13860	13861	13863	13864	13865	13866	13867	13869
	13870	13871	13872	13874	13875	13877	13879	13880	13881
	13882	13926	13929	13976	14066	14497	14612	14696	14739
	14765	14769	14910	15003	15058	15548	15640	15688	15797
	15861	15974	16020	16169	16289	16308	16369	16421	16493
	16495	16856	16928	17166	17288	17319	17386	17387	17389

17391	17392	17393	17394	17395	17396	17397	17398	17399
17401	17402	17404	17406	17407	17408	17411	17414	17415
17416	17417	17418	17419	17421	17422	17423	17424	17426
17427	17428	17429	17430	17431	17432	17433	17434	17435
17436	17437	17438	17439	17441	17442	17444	17445	17446
17448	17451	17452	17453	17454	17455	17456	17459	17463
17464	17465	17466	17467	17468	17469	17470	17471	17472
17473	17474	17475	17476	17479	17481	17482	17483	17484
17486	17488	17489	17490	17492	17494	17498	17500	17501
17502	17503	17504	17506	17507	17508	17509	17510	17513
17514	17516	17517	17518	17519	17520	17521	17522	17523
17524	17525	17526	17527	17610	17611	17613	17614	17617
17618	17619	17620	17622	17623	new ST1	new ST2	new ST3	new ST4
new ST5	new ST6	new ST7	new ST8	new ST9				

Supplementary Table S2. NG-MAST genogroups and associated NG-MAST sequence types, Germany, 2014-2017 (n = 1220).

Genogroup (no. of isolates)	Predominant sequence type (no. of isolates)	Other sequence types (no. of isolates)
G2400 (83)	2400 (29)	9184 (28), 10789 (5), 4943 (4) 8503 (2), 9873 (2), 17478 (2), 17487 (2), 5656 (1), 6360 (1), 7221 (1), 7437 (1), 8115 (1), 13876 (1), 13976 (1), 17448 (1), 17466 (1), 17518 (1)
G1407 (82)	1407 (29)	3779 (11), 2212 (4), 3378 (4), 5533 (4), 4706 (3), 10082 (3), 12280 (3), 12728 (3), 4269 (2), 18193 (2), 4925 (1), 5570 (1), 5622 (1), 9153 (1), 13863 (1), 13879 (1), 17387 (1), 17401 (1), 17404 (1), 17407 (1), 17408 (1), 17622 (1)
G5441 (76)	5441 (47)	13489 (17), 11746 (3), 16819 (2), 12821 (1), 13939 (1), 15688 (1), 16928 (1), 17418 (1), 17426 (1), 17439 (1)
G25 (G51) (68)	25 (37)	3346(5), 51(4), 6701 (3), 273 (2), 5341 (2), 5384 (2), 13868 (2), 17505 (2), 356 (1), 615 (1), 1464 (1), 3519 (1), 10800 (1), 12726 (1), 17416 (1), 17433 (1), 17508 (1)
G2992 (67)	2992 (55)	3558 (2), 5049 (2), 4189 (1), 4954 (1), 5119 (1), 6955 (1), 9249 (1), 9925 (1), 16493 (1), 17519 (1)
G10557 (G7072) (65)	10557 (28)	13878 (15), 7072 (5), 13876 (4), 17450 (3), 17409 (2), 17491 (2), 13869 (1), 17396 (1), 17398 (1), 17421 (1), 17451 (1), 17492 (1)
G11461 (44)	11461 (28)	14764 (11), 17515 (3), 14696 (1), new ST6 (1)
G5624 (41)	5624 (39)	13875 (1), 13926 (1)
G387 (40)	387 (30)	17388 (4), 5743 (3), 1128 (1), 1152 (1), 17517 (1)
G359 (31)	359 (28)	10784 (2), 1929 (1)
G17420 (26)	17420 (13)	16076 (11), 17471 (1), 17507 (1)
G2318 (21)	2318 (13)	10386 (3), 12737 (2), 14051 (2), 11961 (1)
G4186 (G9909) (19)	4186 (10)	10121 (4), 17485 (3), 9909 (2)
G9208 (17)	9208 (16)	15640 (1)
G5793 (15)	5793 (14)	12721 (1)
G225 (14)	225 (13)	17486 (1)
G1405 (12)	1405 (12)	

Supplementary Table S3. AMR distribution among NG-MAST genogroups, Germany, 2014-2017 (n = 1220).

Genogroup (no. of isolates)	Penicillin				Azithromycin				Cefixime			Ceftriaxone			Ciprofloxacin				Beta-lactamase		
	S	I	R	M	S	I	R	M	S	R	M	S	R	M	S	I	R	M	Neg.	Pos.	M
G2400 (83)		82	1		5	25	8		83			83					83		81		2
G1407 (82)		73	9		26	42	14		81	1		82					82		74	1	7
G5441 (76)	11	63	2		73	2	1		76			76			72		4		65	2	9
G25 (G51) (68)	4	27		1	67			1	67		1	67		1	65	2		1	63		5
G2992 (67)	5	62			43	23	1		67			67			65		2		64		3
G10557 (G7072) (65)		62	2	1	64			1	55	9	1	64		1			64	1	61		4
G11461 (44)		44			44				44			44			43		1		44		
G5624 (41)		13	28		35	6			41			41					41		13	26	2
G387 (40)	4				4				4			4			4				34		6
G359 (31)		3	1		27	4			31			31			3		1		3	1	
G17420 (26)		26			26				26			26					26		26		
G2318 (21)	1	2			2		1		21			21			1		2		2		1
G4186 (G9909) (19)	2	17			19				19			19			19				19		
G9208 (17)	1	16			14	2	1		17			17			15		2		17		
G5793 (15)		5	1		15				15			15			15				3	1	2
G225 (14)		3	11		11	3			14			14			1		13		1	4	
G1405 (12)	1	11			12				12			12					12		12		

Supplementary Table S4. Antibiotic susceptibility change across *N. gonorrhoeae* phylogenetic trees.

		Cefixime			Azithromycin		
		2 ln BF[§]	Mean	95% HPD[&]	2 ln BF[§]	Mean	95% HPD[&]
Phylogeny	*porB*	Inf*	1.414	[1-3]	Inf*	5.658	[4-9]
	tbpB	Inf*	1.464	[1-3]	Inf*	5.912	[4-9]

[§] BF: Bayes factor. 2 ln BF > 10 was considered as decisive support for phenotpye distribution change across the phylogeny.
[&] HPD: highest posterior density.
* Inf: Infinity. The model with at least one change is always preferred: it is decisively supported by the analysis.
